# Trophic Dynamics of Mercury in the Baltic Archipelago Sea Food Web: The Impact of Ecological and Ecophysiological Traits

**DOI:** 10.1021/acs.est.2c03846

**Published:** 2022-08-03

**Authors:** Riikka K. Vainio, Veijo Jormalainen, Rune Dietz, Toni Laaksonen, Ralf Schulz, Christian Sonne, Jens Søndergaard, Jochen P. Zubrod, Igor Eulaers

**Affiliations:** † Department of Biology, University of Turku, FI-20014 Turku, Finland; ‡ Department of Ecoscience, Aarhus University, Frederiksborgvej 399, Postbox 358, DK-4000 Roskilde, Denmark; § iES Landau, Institute for Environmental Sciences, University of Koblenz-Landau, Fortstrasse 7, DE-76829 Landau, Germany; ∥ Zubrod Environmental Data Science, Friesenstrasse 20, DE-76829, Landau, Germany; ⊥ Norwegian Polar Institute, FRAM Centre, Postboks 6606 Stakkevollan, NO-9296 Tromsø, Norway

**Keywords:** stable isotopes, food web magnification factor, biomagnification factor, trophic position, Hg, food chain

## Abstract

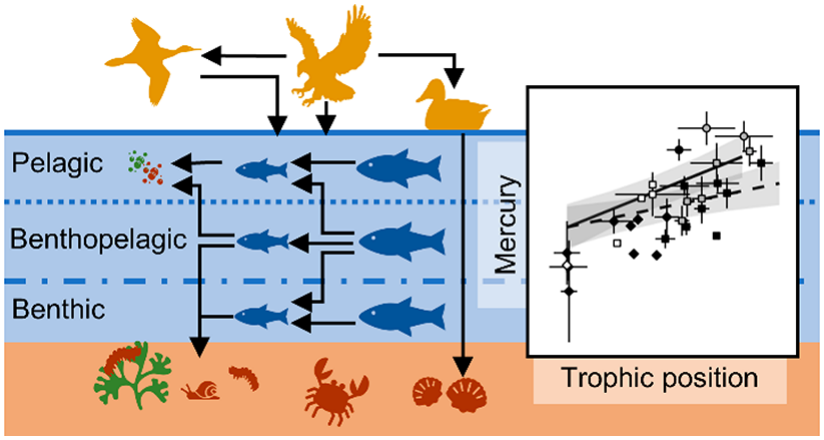

We investigated trophic dynamics of Hg in the polluted Baltic Archipelago Sea using established trophic magnification (TMFs) and biomagnification factors (BMFs) on a comprehensive set of bird, fish, and invertebrate species. As different ecological and ecophysiological species traits may affect trophic dynamics, we explored the effect of food chain (benthic, pelagic, benthopelagic) and thermoregulatory strategy on trophic total Hg (THg) dynamics, using different approaches to accommodate benthopelagic species and normalize for trophic position (TP). We observed TMFs and most BMFs greater than 1, indicating overall THg biomagnification. We found significantly higher pelagic TMFs (3.58–4.02) compared to benthic ones (2.11–2.34) when the homeotherm bird species were excluded from models, but not when included. This difference between the benthic and pelagic TMFs remained regardless of how the TP of benthopelagic species was modeled, or whether TMFs were normalized for TP or not. TP-corrected BMFs showed a larger range (0.44–508) compared to BMFs representing predator–prey concentration ratios (0.05–82.2). Overall, the present study shows the importance of including and evaluating the effect of ecological and ecophysiological traits when investigating trophic contaminant dynamics.

## Abstract 2

Ecophysiological and ecological traits impact the output of established models for trophic contaminant dynamics, potentially having consequences for regulatory decisions based thereon.

## 1 Introduction

Trophic dynamics of contaminants that show biomagnification potential have traditionally been investigated using trophic magnification factors (TMFs) and biomagnification factors (BMFs).^1^ TMFs measure the average increase in contaminant concentration per trophic level, often proxied by bulk stable nitrogen isotopes (δ^15^N) due to their characteristic trophic enrichment.^2^ BMFs in turn measure the increase in contaminant concentration from prey to predator. However, species’ ecological and ecophysiological traits, such as food chain origin, trophic position, and thermoregulatory strategy, may cause variation in the underlying ecological and contaminant variability when studying the trophic dynamics of contaminants, highlighting the need for trait-based modeling of contaminant dynamics.^3^ Nonetheless, risk assessment modeling, typically using such TMFs and BMFs, does not commonly take into account such species traits nor their potential impact on modeling outcomes.

Ecotoxicological studies on marine systems have indicated that contaminant loadings and pathways differ between benthic and pelagic food chains due to for example differences in bioavailability.^4−8^ Moreover, often food chains are assumed linear and discrete, while generalist species often feed on prey residing at multiple trophic positions or in different food chains, which may in fact be closely interlinked (for example benthopelagic coupling). Aside from all ecological species characteristics complicating practical discretization of food chains and their composing trophic positions, species exhibit ecophysiological traits, such as thermoregulation and respiration, that influence contaminant bioaccumulation and biomagnification. Indeed, species-differences in thermoregulatory strategy, resulting in differences in metabolism and energy requirements, can cause homeotherm species to accumulate higher contaminant concentrations than poikilotherms.^9−11^ In fact, BMFs are currently approached with multiple different formulas,^12^ some formulas normalizing for trophic position differences for the predator–prey pairs, while some do not. Even so, such potential impacts of different species-traits on BMFs, or TMFs, have not been consequently reported or robustly investigated for their importance when studying food web dynamics of contaminants.

The Baltic Sea is particularly susceptible to pollution, due to its geophysical location and hydrological properties^13^ and has a well-known legacy for persistent contaminants that typically show biomagnification.^14,15^ Among a plethora of hazardous substances found in the Baltic Sea is mercury (Hg^14^), for which emissions have increased dramatically since the Industrial Revolution.^16^ Mercury is environmentally persistent, and concentrations are found to be elevated in most Baltic Sea basins^17^ and readily bioavailable to the lower functional groups of the food web especially as organic methylmercury (MeHg), resulting in Hg bioaccumulation in higher trophic species and ultimately Hg biomagnification through food chains.^18^

Here we investigate the trophic dynamics of total Hg (THg) in benthic and pelagic food chains of the Baltic Archipelago Sea, using established TMFs and BMFs on a comprehensive set of species (*n* = 30), across different functional groups, that are key in this low diversity ecosystem. To investigate the impact of different species traits on trophic magnification models, we explored the effect of two ecological traits, i.e., the trophic position food chain origin (benthic, pelagic, benthopelagic) and one ecophysiological trait, i.e., thermoregulatory strategy (homeotherm, poikilotherm) on trophic THg dynamics, using different approaches to accommodate benthopelagic species and normalizing for trophic position (TP).

## 2 Materials and Methods

### 2.1 Sample Collection

**Figure 1:**
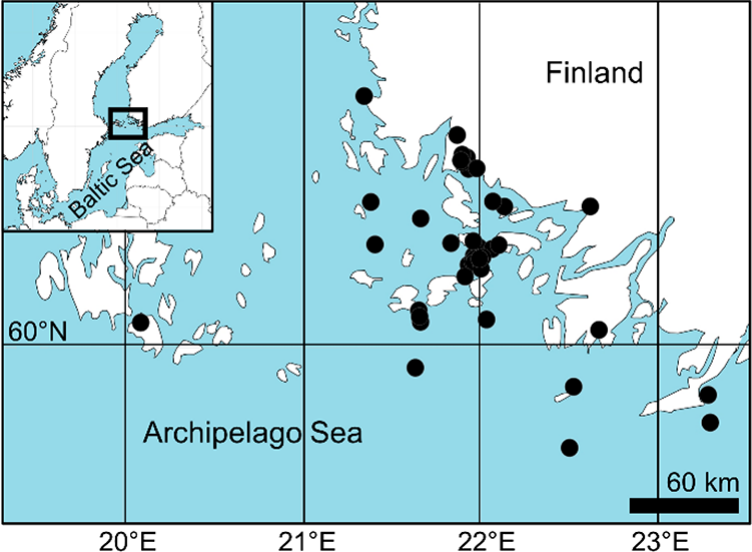
Map of the sampling locations.

We collected samples of birds, fish, and invertebrates during the period 2017–2019, except for the extended time frame (2013–2019) for white-tailed eagle (*Haliaeetus albicilla)*, in the Archipelago Sea, a Baltic Sea basin at the southwestern coast of Finland (Figure 1).

The Archipelago Sea is one of the largest archipelagos and is a shallow brackish water ecosystem with low salinity, inhabited by both marine and freshwater species. Information on the sampling methods, collection times, tissues used for chemical analysis, and use of composite versus individual samples are presented in Table S1. We measured body length for invertebrates and fish, and individual fish mass was recorded when possible (Table S2). All birds were dissected for muscle. From larger fish species the dorsal fillet was dissected, while from smaller species the intestinal tract was removed, except from lesser sand eel (*Ammodytes tobianus*) and sand goby (*Pomatoschistus minutus*), before homogenization of the entire individual using a shear mixer. We pooled individual invertebrates sampled at the same location to constitute a composite sample with sufficient mass for chemical analysis. We transported all samples under cool and dark conditions during field collection and stored them at −20 °C prior to sample preparation for chemical analysis, prior to which samples were freeze-dried at −50 °C, gravimetrically determined for its dry matter, and finally homogenized using pestle and mortar.

### 2.2 Mercury Analysis

The analysis for total mercury was performed using a direct mercury analyzer (DMA-80, Milestone, Italy) at the accredited Trace Element Laboratory at Department of Ecoscience, Aarhus University, following the US-EPA Method 7473^19^ during the period 2019–2021. The analytical quality was controlled by concurrently analyzing aqueous standard solutions (10 ng and 100 ng of Hg, prepared from 1000 ± 4 mg L^–1^ stock solution, Sigma-Aldrich, Switzerland), procedural blanks, duplicates, and Certified Reference Material (DORM-4; fish protein from the National Research Council, Canada; 0.412 ± 0.036 μg Hg g^–1^ dry weight, dw). Measurements were subsequently corrected for instrumental drift using results from the aqueous 10 and 100 ng standards applied to the low- and high-concentration cell, respectively (drift was always < 10%). All samples were corrected for concurrent blanks (<0.1 ng). The measured recovery long-term percentage of the DORM-4 was 102 ± 6% (mean ± SD; *n* = 284 during 2019–2021). This shows satisfactory accuracy and a precision (extended analytical uncertainty) (2 SD) of ca. 10% for the analyses. The laboratory is accredited by the Danish Accreditation Fond DANAK following ISO 17025 to DMA-80 analyses of Hg in biota with a detection limit of 0.001 μg g^–1^ dw. As part of the laboratory QA/QC, the lab participates twice a year in the international laboratory performance study program QUASIMEME (www.quasimeme.org). The participation has proved excellent long-term measurement accuracy and precision (*n* = 15 during 2017–2021; assigned concentration from 0.009 to 0.931 μg g^–1^ dw; *Z*-scores from −1.0 to 0.7 with a mean of −0.1).

### 2.3 Stable Isotope Analysis

The stable isotope analysis was carried out at the Stable Isotope Lab of the University of Koblenz-Landau (Germany). Ratios of stable nitrogen isotopes (^15^N:^14^N) were determined on a freeze-dried and homogenized subsample (mean ± SD: 1.48 ± 0.06 mg) using a Flash 2000 HT elemental analyzer coupled via a ConFlo IV interface to a Delta V Advantage isotope ratio mass spectrometer (all Thermo Fisher Scientific, Bremen, Germany). The stable nitrogen isotope values are reported following best practices suggested by Bond and Hobson^20^ and are relative to their respective international measurement standard of atmospheric N_2_. Internal reference material (casein) was measured concurrently in duplicate every 10 samples, revealing an imprecision (±SD) ≤ 0.06 ‰.

### 2.4 Data Analysis

To investigate the effect of ecological and ecophysiological traits on trophic magnification of THg, we categorized the study species, based on their diet, for their food chain (18 benthic, five pelagic, and seven benthopelagic species) and thermoregulatory strategy (three homeotherm, 27 poikilotherm). Rather than relying on the empirical stable isotope values we used literature on the studied species (see Supporting Information for full reference list) to define species to be pelagic or benthic, when mainly feeding in a food chain depending on pelagic or benthic basal energy sources, respectively, or to be benthopelagic when feeding in both.

We calculated the TP of an individual of a benthic and pelagic species following Post:^21^
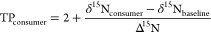
1where the trophic enrichment factor (TEF) Δ^15^N was set at 3.40, as recommended by Borgå et al.,^1^ and where δ^15^N_baseline_ is the δ^15^N value for the chosen benthic or pelagic primary consumer species (assumed to feed at TP = 2), that is, *Gammarus* spp. and zooplankton, respectively for benthic and pelagic species. Primary consumer species are preferred as food chain baselines as primary producers’ δ^15^N values can show large spatiotemporal variability due to seasonal variation in growth rate and different nutrient inputs in different locations.^21^ Due to this variability, we omitted primary producers from the trophic magnification models (See Supporting Information for more on the primary producers).

The calculation of TPs for benthopelagic species can however not follow this one-source model, as mixed feeding on both benthic and pelagic prey comes along with dependency on two δ^15^N baselines. Because of this, we calculated the TP for individuals of benthopelagic species following two approaches. In the first approach, we assumed that the benthopelagic species feeds entirely in either benthic or pelagic habitat, that is, from one basal energy source, and therefore the above-described one-source TP calculation was used for both food chains separately. In the second approach, we used a two-source model, assuming that the benthopelagic species feeds in both benthic and pelagic habitats, calculating the TP following the two-source formula outlined by Post:^21^
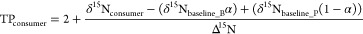
2where δ^15^N_baseline_B_ and δ^15^N_baseline_P_ are the δ^15^N values of benthic and pelagic baselines, respectively. As the actual proportions in which benthopelagic species feed from benthic and pelagic food chains are unknown, we set the proportion of feeding from the first food chain to α = 0.50.

For birds, a TEF of Δ^15^N = 2.4 is more appropriate to represent trophic enrichment between bird tissue and that of their prey.^22^ Hence, we calculated TPs for benthic common eiders in the one-source model following Hop et al.:^9^
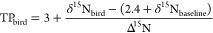
3while TPs for the benthopelagic white-tailed eagle and great cormorant were calculated after adaptation from Hop et al.^9^ and Post:^21^
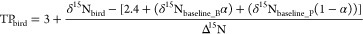
4with α = 0.50. As the δ^15^N signal of whole fish and muscle tissue can show small differences, the TP estimated for whole fish might differ 0–0.6 trophic levels compared to TP determined from the muscle tissue.^23,24^

We calculated TMFs, also referred to as food web magnification factors (FWMFs), separately for the benthic and pelagic food chains, using both one-source and two-source models due to the included benthopelagic species. We used a linear mixed model with log_10_[THg dw] as dependent variable, TP or δ^15^N as independent variable, and species as a random variable due to unbalanced sample sizes across the studied species. Using the slope *b* of each fitted model we calculated the TMF following
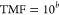
5The slope of the regression measures the increase in THg concentrations with the calculated TPs across the food chain, while the intercept represents the biological and environmental factors that determine the input of THg into the food chain at its base.^1^ We used dw concentrations due to smaller variation of wet weight concentrations negatively effecting the reliability of the models. To assess the impact of homeotherm species on the trophic magnification models, we repeated all models excluding the three bird species. To compare the goodness of fit for the models, we calculated coefficients of determination for each model according to Nagawa et al.:^25^ marginal *R*_m_^2^, which represents the total variance explained by the fixed effects, and conditional *R*_c_^2^, representing proportion of variance explained by both fixed and random effects. Differences in the slopes and intercepts between the benthic and pelagic food chain models were tested using a Welch’s *t* test.

Finally, to assess the effect of normalizing BMFs for TP, we calculated BMFs for all likely predator–prey pairs, based on literature recordings of the diet of the species (see Supporting Information), with normalization for TP:
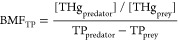
6and without:
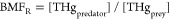
7The difference between the values produced by the two BMF approaches was calculated for each predator–prey pair by taking the absolute value of the subtraction of the BMF_TP_ and BMF_R_. Due to consisting of only one individual, we omitted Atlantic salmon from the BMF calculations. Full BMF results with Atlantic salmon can be found in Tables S4–S7.

## 3 Results

**Figure 2:**
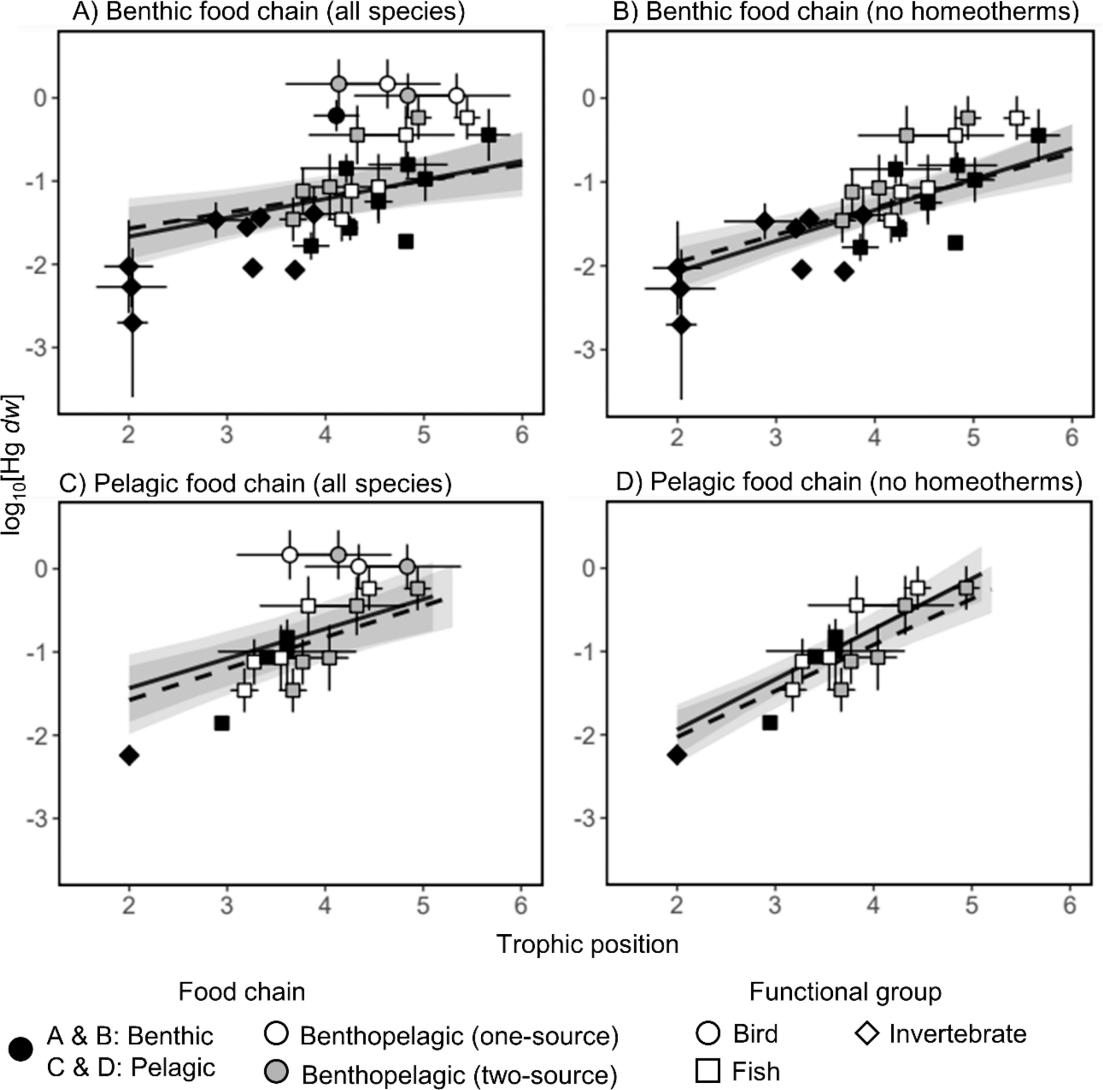
Linear regressions between log_10_[THg dw] (μg g^–1^) and trophic position for (A) benthic habitat (all species), (B) benthic habitat (no birds), (C) pelagic habitat (all species), and (D) pelagic habitat (no birds). The solid lines are for regression of the one-source model (eqs 1 and 3), while the dashed lines are for the two-source model (eqs 2 and 4).

Summary statistics for δ^15^N, TP, and THg for all studied species are given in Table 1, and the extended summary statistics are given in Table S3. All TMFs exceeded one, indicating food web magnification (Figure 2; Table 2).

**Table 1:** Mean ± SD Values for δ^15^N, TP, and THg Concentrations (μg g^–1^ ww and dw) for the Studied Species, Grouped According to Their Food Chain^a^

scientific name	common name	*n*	*δ*^15^N	TP	Hg dw	Hg ww
Pelagic						
*Ammodytes tobianus*	lesser sand eel	1*	+10.73	2.95	0.014	0.0027
*Clupea harengus*	herring	10	+13.00 ± 2.39	3.61 ± 0.70	0.15 ± 0.17	0.035 ± 0.041
*Coregonus albula*	vendace	2	+12.29 ± 0.22	3.41 ± 0.06	0.087 ± 0.013	0.018 ± 0.003
*Salmo salar*	Atlantic salmon	1	+13.00	3.61	0.15	0.055
zooplankton		3*	+7.52 ± 0.14	2.00 ± 0.04	0.0059 ± 0.0015	0.0006 ± 0.0002
Benthic						
*Somateria mollissima*	common eider	13	+10.32 ± 0.79	4.11 ± 0.23	0.67 ± 0.31	0.17 ± 0.09
*Abramis brama*	common bream	6	+13.80 ± 1.37	4.84 ± 0.40	0.17 ± 0.06	0.042 ± 0.018
*Gobius niger*	black goby	5	+11.80 ± 0.27	4.25 ± 0.08	0.028 ± 0.0087	0.0060 ± 0.0019
*Gymnocephalus cernua*	Eurasian ruffe	10	+14.40 ± 0.69	5.01 ± 0.20	0.13 ± 0.08	0.026 ± 0.017
*Myoxocphalus quadricornis*	fourhorn sculpin	5	+16.659 ± 0.75	5.66 ± 0.22	0.45 ± 0.38	0.083 ± 0.055
*Neogobius melanostomus*	round goby	10	+10.45 ± 0.64	3.85 ± 0.19	0.018 ± 0.005	0.0044 ± 0.0020
*Pomatoschistus minutus*	sand goby	1*	+13.73	4.82	0.019	0.0045
*Rutilus rutilus*	common roach	10	+11.67 ± 1.58	4.21 ± 0.46	0.15 ± 0.06	0.035 ± 0.014
*Zoarces viviparus*	viviparous eelpout	10	+12.78 ± 0.49	4.54 ± 0.14	0.066 ± 0.036	0.015 ± 0.008
*Gammarus spp.*		6*	+4.15 ± 0.84	2.00 ± 0.25	0.018 ± 0.026	0.0037 ± 0.0050
*Idotea spp.*		4*	+4.25 ± 1.22	2.03 ± 0.36	0.0060 ± 0.0035	0.0016 ± 0.0010
*Macoma balthica*	Baltic clam	4*	+8.70 ± 0.29	3.34 ± 0.09	0.037 ± 0.006	0.0074 ± 0.0013
*Mytilus edulis*	blue mussel	9*	+7.16 ± 1.40	2.88 ± 0.41	0.037 ± 0.013	0.0059 ± 0.0023
*Palaemon adpersus*		1	+9.90	3.69	0.0086	0.0019
*Palaemon elegans*		1*	+8.44	3.26	0.0091	0.0022
*Rhithropanopeus harrisii*		1*	+8.24	3.20	0.028	0.0071
*Saduria entomon*		2*	+10.55 ± 0.79	3.88 ± 0.23	0.05 ± 0.03	0.016 ± 0.014
*Theodoxus fluviatilis*		3*	+4.29 ± 0.53	2.0 ± 0.16	0.0048 ± 0.0050	0.0021 ± 0.0022
Benthopelagic						
*Haliaeetus albicilla*	white-tailed eagle	7	+12.09 ± 1.83	3.64 ± 0.54 (P)	1.9 ± 1.7	0.51 ± 0.34
				4.63 ± 0.54 (B)		
				4.13 ± 0.54 (T)		
*Phalacrocorax carbo*	great cormorant	6	+14.48 ± 1.86	4.34 ± 0.55 (P)	1.3 ± 1.0	0.35 ± 0.27
				5.33 ± 0.55 (B)		
				4.84 ± 0.55 (T)		
*Coregonus lavaretus*	common whitefish	2	+11.85 ± 0.16	3.27 ± 0.05 (P)	0.084 ± 0.049	0.019 ± 0.011
				4.26 ± 0.05 (B)		
				3.77 ± 0.05 (T)		
*Esox lucius*	northern pike	9	+15.84 ± 0.46	4.45 ± 0.13 (P)	0.68 ± 0.43	0.15 ± 0.09
				5.44 ± 0.13 (B)		
				4.9 ± 0.13 (T)		
*Gasterosteus aculeatus*	three-spined stickleback	6	+11.52 ± 0.47	3.18 ± 0.14 (P)	0.039 ± 0.018	0.010 ± 0.004
				4.17 ± 0.14 (B)		
				3.67 ± 0.14 (T)		
*Osmerus eperlanus*	European smelt	10	+12.78 ± 0.65	3.55 ± 0.22 (P)	0.12 ± 0.08	0.024 ± 0.017
				4.54 ± 0.19 (B)		
				4.04 ± 0.19 (T)		
*Perca fluviatilis*	European perch	10	+13.73 ± 1.68	3.83 ± 0.49 (P)	0.48 ± 0.38	0.095 ± 0.070
				4.82 ± 0.49 (B)		
				4.32 ± 0.49 (T)		

a The TPs for benthopelagic species are calculated to comply with either the pelagic (P) or benthic (B) food chain baseline species both using a one-source model (eqs 1 and 3), or to accommodate both baseline species, using a two-source model (T) (eqs 2 and 4). Pooled samples are marked with an asterisk (∗).

**Table 2:** Statistical Output of Linear Models and the Resulting TMFs for THg (μg g^–1^ dw) in the Archipelago Sea, Accommodating Different Configurations to Test the Impact of the Species Traits Trophic Position, Food Chain Origin, and Thermoregulatory Strategy^a^

model	*n*	*R*_m_^2^	*R*_c_^2^	*p*	intercept (SE)	slope (SE)	TMF
Pelagic Food Chain							
one-source	70	0.14	0.78	<0.01	–2.15 (0.34)	0.36 (0.08)	2.27
one-source (no birds)	54	0.55	0.70	<0.01	–3.15 (0.31)	0.60 (0.09)	4.02
two-source	70	0.26	0.76	<0.01	–2.33 (0.32)	0.38 (0.08)	2.38
two-source (no birds)	54	0.59	0.73	<0.01	–3.13 (0.30)	0.55 (0.08)	3.58
δ^15^N^b^	70	0.11	0.80	<0.01	–2.14 (0.36)	0.10 (0.03)	1.26
δ^15^N (no birds)^b^	54	0.55	0.70	<0.01	–3.28 (0.33)	0.18 (0.06)	1.51
Benthic Food Chain							
One-source	154	0.11	0.80	<0.01	–2.12 (0.29)	0.23 (0.07)	1.69
One-source (no birds)	125	0.42	0.71	<0.01	–2.81 (0.25)	0.37 (0.06)	2.34
Two-source	154	0.06	0.82	<0.01	–1.96 (0.30)	0.19 (0.07)	1.56
Two-source (no birds)	125	0.29	0.71	<0.01	–2.61 (0.28)	0.32 (0.07)	2.11
δ^15^N^b^	154	0.08	0.81	<0.01	–1.87 (0.25)	0.06 (0.02)	1.15
δ^15^N (no birds)^b^	125	0.46	0.71	<0.01	–2.52 (0.21)	0.11 (0.02)	1.28

a The TP of benthopelagic species was estimated using only pelagic or benthic baseline species (one-source model, eqs 1 and 3) or both (two-source model, eqs 2 and 4). *n* = sample size, *R*_m_^2^ = marginal coefficient of determination, *R*_c_^2^ = conditional coefficient of determination, *p* = significance for the slope, SE = standard error.

b Note that the TMFs for the models using δ^15^N represent THg biomagnification per unit increase of δ^15^N (‰) instead of per trophic level.

We did not observe statistically significant differences between the pelagic one- and two-source model regardless of whether homeotherms were included or not (−0.38 ≤ *t* ≤ 0.44; 0.66 ≤ *p* ≤ 0.97). Similarly, we did not observe differences between benthic one- and two-source models with or without the homeotherms (−0.54 ≤ *t* ≤ 0.47; 0.59 ≤ *p* ≤ 0.71). The slopes or intercepts did not differ between the respective benthic models or the pelagic two-source models with or without the homeotherms (−0.21 ≤ *t* ≤ 1.76; 0.08 ≤ *p* ≤ 0.83; pelagic two-source: −1.57 ≤ *t* ≤ 1.76; 0.08 ≤ *p* ≤ 0.12). However, there was a difference between both slopes (*t*_120_ = −2.06; *p* = 0.04) and intercepts (*t*_120_ = 2.19; *p* = 0.03) for the pelagic one-source models with and without the homeotherms. We did not find differences between the benthic and pelagic models, irrespective of the one- or two-source approach, when homeotherms were included (−1.73 ≤ *t* ≤ 0.82; 0.09 ≤ *p* ≤ 0.96). There were also no differences between the model intercepts for the benthic and pelagic food chains, neither using a one-source model (*t*_175_ = 0.85; *p* = 0.40) nor a two-source one (*t*_175_ = 1.28; *p* = 0.20) when homeotherms were excluded.

However, in the models excluding homeotherm bird species, there was a significant difference in the slopes between the pelagic and benthic one-source (*t*_175_ = −2.24; *p* = 0.03) and two-source models (*t*_175_ = −2.20; *p* = 0.03). Regression slopes showed to be steeper in the pelagic food chain in both cases (Table 2). There was also a significant difference between benthic and pelagic slopes (*t*_175_ = −2.24; *p* = 0.03) as well as between the intercepts (*t*_175_ = −1.94; *p* = 0.05) of the TMF_δ15N_ when excluding birds. Again, the slope showed to be steeper in the pelagic food chain, while intercept was higher in the benthic food chain (Table 2). Omitting birds from the models increased the explanatory power (*R*_m_^2^; Table 2) compared to the respective models including these homeotherm species.

**Figure 3:**
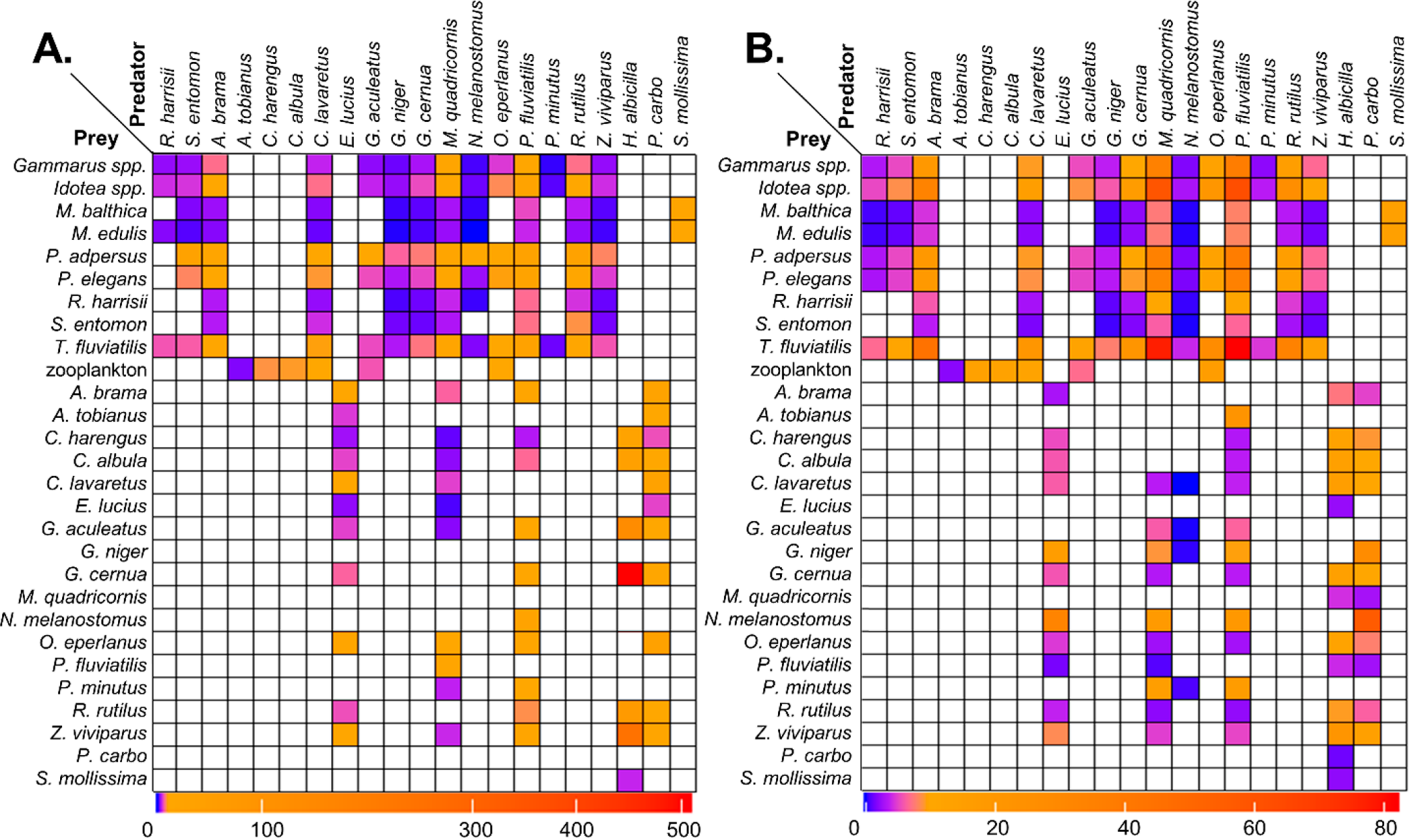
Heatmap of biomagnification factors (BMFs) for relevant predator–prey pairs (A) normalized for TP (eq 6) or (B) not (eq 7). Negative BMF values were removed.

All obtained BMFs calculated using eqs 6 and 7 are given in Tables S4–S7. We observed 95.8% of BMF_TP_ (eq 6) and 91.5% of BMF_R_ (eq 7) to be greater than 1, indicating overall biomagnification. For homeotherms, we observed a range of BMF_TP_ = 4.40–508 and BMF_R_ = 1.48–56.8, while for poikilotherms BMF_TP_ = 0.44–33.5, and BMF_R_ = 0.19–82.2, values of BMF_TP_, but not BMF_R_ being higher for homeotherms than poikilotherms. For pelagic predators, BMF_TP_ = 1.61–9.35 and BMF_R_ = 2.11–14.4, for benthic predators BMF_TP_ = 0.44–30.9 and BMF_R_ = 0.19–77.6, and for benthopelagic predators BMF_TP_ = 1.61–508 and BMF_R_ = 1.48–82.2, with highest BMFs in both cases found in the benthopelagic species, followed by benthic and pelagic species. Higher variation was observed for the obtained BMF_TP_ (eq 6; mean ± SD = 14.5 ± 45.4; range, 0.44–508; Figure 3A), than for BMF_R_ (eq 7; 11.2 ± 13.4; 0.19–82.2; Figure 3B). There was high variability in the difference between BMF_TP_ and BMF_R_ for the same predator–prey pair (mean ± SD = 11.5 ± 44.0; range, 0.004–494.2), the difference being greater than 10 in 19.9% of the pairs. The denominator in eq 6, that is, the normalization for TP, also was a cause for 9.6% of the predator–prey pairs negative BMF_TP_ (removed from Figure 3A) due to the predator’s TP being lower than that of its prey (see Discussion in the Supporting Information).

## 4 Discussion

We investigated the impact of two ecological traits, trophic position and food chain origin, and an ecophysiological trait, thermoregulatory strategy, on established risk assessment modeling approaches, that is, TMFs and BMFs, to study trophic contaminant dynamics of mercury in the Baltic Archipelago Sea. Overall, TMFs and BMFs showed THg biomagnification to be affected by distinguishing food chains and the presence of homeotherm species. We also found most BMFs to be highly variable between different predator–prey pairs and formulas of calculation.

For both the benthic and pelagic food chains large positive TMF values showed strong THg food web magnification. The observed TMF_δ15N_ values for the pelagic food chain (1.28–1.51) were similar to the TMF_δ15N_ value of 1.50 reported for a pelagic food chain of zooplankton, Mysis spp., and herring in the Baltic Sea.^26^ The trophic magnification slopes on δ^15^N in our study (0.06–0.18) also matched the average of all sites (mean ± SD: 0.16 ± 0.11), temperate sites (0.17 ± 0.10), and marine coastal sites (0.19 ± 0.08) evaluated in a worldwide meta-analysis by Lavoie et al.^27^ The number of trophic levels (TP_top predator_ – TP_baseline_) in both benthic (3.7) and pelagic (2.9) food chains in our study included more trophic levels than the studies (mean ± SD 1.7 ± 0.7) included in the same meta-analysis.^27^ However, we found the TMFs derived separately for the benthic and pelagic food chains of the Archipelago Sea to reveal higher trophic magnification of THg in the pelagic food chain when excluding birds. This result is robust as it remained independently of how TPs of benthopelagic species were modeled. Similar to our study, higher TMFs in the pelagic food chain compared to those in the benthic one have been reported in the Gulf of St. Lawrence, Canada,^4^ and Santos continental shelf, Brazil.^6^ Pelagic pathways have been suggested to be more efficient in transferring highly bioaccumulative and biomagnifying MeHg compared to transfer from sediments, MeHg being more readily bioavailable in the pelagic food chain.^28,29^ Although studies have shown both inorganic and organic species of Hg being capable of being transferred through the diet and assimilated into organisms’ tissue, both the rate of absorption and assimilation are higher for MeHg than inorganic species of Hg,^30^ leading to higher rates of both bioaccumulation and biomagnification for MeHg, possibly contributing to the higher TMF observed for the pelagic food chain. Also, although the THg concentrations at the base of both benthic and pelagic food chains of our study were similar and dietary pathways dominate the Hg uptake in higher trophic levels (for example refs (30 and 31)), possible additional uptake of Hg through bioconcentration may partly explain the difference in TMFs between the benthic and pelagic food chains. These possibly different pathways of Hg into the benthic and pelagic food chain and proportions of MeHg should be further investigated to unravel their effect on the trophic dynamics of Hg. However, trophic contaminant dynamics between food chains are system-specific due to differences in for example physiochemical properties of the environment, and thus inherent variability in the resulting contamination pathways. Our results demonstrate the importance of investigating differences in trophic transfer of contaminants between different food chain pathways to better understand the contaminant dynamics.

In the present study, we modeled the TPs of benthopelagic species in two ways: assuming linearity of the food chain with its composing species feeding from only one basal energy source and allowing for the possibility of cross-food chain feeding depending on two basal energy sources. Often TP and trophic magnification models based thereon accommodate only one baseline species. However, most generalist and mobile species forage in different environments and food chains. Prey-switching can happen due to availability and ontogeny, and individuals can move between environments between seasons and stages of life cycle.^32−34^ Recently, studies have investigated the coupling of benthic and pelagic systems^35^ suggesting that benthic and pelagic habitats are coupled more closely in various ways than previously assumed.^36^ The modeling approach (one- or two-source model) for the benthopelagic species did not affect the TMFs. While our two-source model approach, assuming benthopelagic species feeding 50% in either food chain, is perhaps still not entirely accurate as actual consumer-specific prey proportions would show, it offers a more ecologically realistic solution to accommodate species that show cross-food chain feeding strategies when using bulk stable isotopes for TP estimation. Moreover, applying an analysis for compound-specific stable isotopes^37,38^ may provide additional insights and, perhaps, an even more robust assessment of the trophic dynamics of Hg in the studied food web.

Homeotherm species may accumulate higher concentrations of recalcitrant contaminants, such as Hg, than poikilotherms due to higher energy requirements demanding higher food consumption and thus intake of contaminants.^1,9^ Moreover, as long-lived species, birds bioaccumulate recalcitrant contaminants during their lifetime.^39^ Trophic magnification models including homeotherm birds did not show different trophic magnification between the benthic and pelagic food chain, in sharp contrast to the models excluding homeotherms. Moreover, all trophic magnification models without homeotherm species showed better statistical fit (*R*_m_^2^) compared to models including homeotherms. However, omitting the homeotherm birds affected TMFs only for the pelagic one-source model, with TMF being higher without the birds. We found THg concentrations in homeotherms to be relatively elevated in comparison to species from other functional groups exhibiting similar TP. Similarly, Wang et al.^10^ modeled MeHg biomagnification in floodplain food webs and found that the regression line for homeotherms had a higher intercept than the one for poikilotherms. While such a comparison should be made with precaution, as the present study cannot provide insight on MeHg dynamics, our findings also seem to suggest that the impact of thermoregulatory strategy on both within food chain and between food chain TMF estimates merits future consideration of designing and comparing trophic contaminant magnification modeling attempts.

More than 90% of all BMFs exceeded 1, further demonstrating THg biomagnification in the Archipelago Sea food web. For BMF_TP_, homeotherms had a larger range and higher maximum values than poikilotherms, while for BMF_R_ ranges of homeotherms and poikilotherms were similar. When comparing BMFs across food chains, the highest BMF_TP_ and BMF_R_ were found for benthopelagic predators, and the lowest ones were found for pelagic predators. The total range of BMFs was large, especially BMF_TP_. This was not only due to differences in THg concentrations between predator and its prey, but also due to a small TP difference: small TP differences are therefore observed to inflate BMF_TP_. Conversely, large differences in δ^15^N-derived TPs may also lead to underestimation of the biomagnification potential. Large disparities in BMFs with and without the normalization for TPs may indicate that the actual diet of the predator includes also other sources of contamination than the prey used for calculating BMFs,^40^ while also other factors, such as disparity in temporality, may affect BMFs. Currently, there seem to be no unbiased BMF estimations as each model (see Franklin^12^) has trade-offs between ecological reality and statistical applicability. Different ecological and physiological factors, such as uncertainties in species feeding ecology and metabolism, also affect BMFs, and cause variability in BMFs of the same contaminants between different studies.^12^ Indeed, our results show that different ecological and ecophysiological species traits, such as the here studied food chain origin, TP, and thermoregulatory strategy, introduce uncertainty in BMF comparisons making comparisons within and across studies more difficult. Our finding of highly variable BMFs supports the recommendation of using TMFs rather than BMFs, as TMFs represent the average biomagnification across the food chain and are less likely to be affected by individual species traits than the BMFs.^41^

A recent risk assessment of THg concentrations on the Baltic Sea^42^ showed the fish and bivalves being at low risk of Hg mediated health effects, while in white-tailed eagles the risk of negative health effects was assessed to be moderate to high. However, the results of the risk assessment should be taken as indicative, as variation in the proportions of highly toxic MeHg between both species and individuals can occur.^42^ Also, climate change mediated increases in runoff combined with other factors, such as shorter icing period, may increase Hg fluxes into the Baltic Sea as well as Hg bioavailability,^43^ altogether potentially increasing the loadings and changing the dynamics of Hg in the food web. Because of these concerns, it is important to understand the role of ecological and ecophysiological traits on trophic contaminant dynamics, as well as the vulnerability of current models used for regulatory decisions. Moreover, the novel functional ecological understanding and comparative model framework of the present study should be further investigated for implications due to Hg speciation dynamics.

## Supporting Information Available

Methods and discussion of primary producer and trophic positions of the species, literature references on the diet of the study species, Tables S1–S7 with additional information on the sampling, summary statistics, and full biomagnification factors (PDF)
