# Supplementary material for: Trophic Dynamics of Mercury in the Baltic Archipelago Sea Food Web: The Impact of Ecological and Ecophysiological Traits
Source: Environ Sci Technol. 2022 Aug 3;56(16):11440–8. doi: 10.1021/acs.est.2c03846 (PMC9387095; doi:10.1021/acs.est.2c03846)
Supplement: Supplementary file 1 — es2c03846_si_001.pdf [file es2c03846_si_001.pdf]

Trophic dynamics of mercury in the Baltic Archipelago Sea food web: the impact of ecological and ecophysiological traits

Riikka K. Vainio[*1], Veijo Jormalainen[1], Rune Dietz[2], Toni Laaksonen[1], Ralf Schulz[3], Christian Sonne[2], Jens Søndergaard[2], Jochen P. Zubrod[3,4], Igor Eulaers*[2,5]

[1] Department of Biology, University of Turku, FI-20014 Turku, Finland

[2] Department of Ecoscience, Aarhus University, Frederiksborgvej 399, DK-4000 Roskilde, Denmark

[3] iES Landau, Institute for Environmental Sciences, University of Koblenz-Landau, Fortstrasse 7, DE-76829 Landau, Germany

[4] Zubrod Environmental Data Science, Friesenstrasse 20, DE-76829, Landau, Germany

[5] Norwegian Polar Institute, FRAM Centre, Postboks 6606 Stakkevollan, NO-9296 Tromsø, Norway

[*] Corresponding authors: Riikka Vainio (rikavai@utu.fi), Igor Eulaers (Igor.Eulaers@npolar.no)

Number of pages (including Cover page and Table of Contents): 18

Tables S1-S7

Table of contents

1. Supplementary methods S1

3. Supplementary discussion S2

5. Equations S5

6. References to support food chain classification S6

Table S2. Body measurements of the study species. S9

Table S3. Summary statistics for *$\delta^{15}N$*, C:N ratio, dry and wet weight Hg concentrations, and dry matter content for the study species S10

Table S4. Biomagnification factors (BMFs) for benthic predator-prey pairs using median, minimum, and maximum dry weight Hg concentrations (per Eq. 1). S11

Table S5. Biomagnification factors (BMFs) for pelagic predator-prey pairs using median, minimum, and maximum dry weight Hg concentrations (per Eq. 1). S13

Table S6. Biomagnification factors (BMFs) for benthic predator-prey pairs using median, minimum, and maximum dry weight Hg concentrations (per Eq. 2). S14

Table S7. Biomagnification factors (BMFs) for pelagic predator-prey pairs using median, minimum, and maximum dry weight Hg concentrations (per Eq. 2). S16

1. Supplementary methods

In addition to the birds, fish, and invertebrates, we collected samples of three benthic primary producers (*Cladophora glomerata*, *Fucus spp.*, *Pilayella/Ectocarpus spp*.) and one pelagic primary producer (phytoplankton). Information on the sampling methods and collection times of the primary producers are presented in Table S1, and summary statistics in Table S3.

3. Supplementary discussion

The $\delta^{15}N$ signal of primary producers can show high temporal and spatial variation [1, 2], while the $\delta^{15}N$ signal of primary consumers integrates the temporal variation in the $\delta^{15}N$ signal of the primary producers [2]. In our data, the $\delta^{15}N$ signals of the primary producers of the benthic habitat were similar to those of primary consumers, *Idotea* spp., *Gammarus spp.*, and *Theodoxus fluviatilis*, despite the primary producers being the first trophic level of the food web. These results highlight the importance of using primary consumers instead of primary producers as the trophic baseline organisms when constructing trophic structures, especially when sampling has not been conducted to account for the temporal variation in $\delta^{15}N$ of the primary producers.

We found high Hg concentrations in phytoplankton, with a mean concentration of more than 10-fold of those found in zooplankton, and similar to those found in herring and salmon. There was a five-fold difference in Hg concentrations between the two phytoplankton samples, values being 0.04 and 0.2 µg/g dw. Similar variation was observed also in the $\delta^{15}N$ signal of the phytoplankton samples, possibly indicative of different input of nutrients and Hg contamination between the two sampling locations. Similar variation was not observed on the zooplankton samples, collected during the same time in same locations, or in *C. glomerata* or *Pilayella/Ectocarpus spp.,* collected at the same date close to the plankton sampling locations. Hg concentrations similar to ours in phytoplankton, with large temporal variation, have been reported in the southern Baltic Sea, with the highest concentrations found towards late autumn due to combination of prevailing species composition and increased input into the environment [3]. As the species composition in our samples is unknown, it is possible that the high concentrations might be due to presence of species with higher bioconcentration of Hg compared to the environment.

Due to the food-chains, instead of being linear constructs, forming complex food-webs, where consumption may occasionally target to a species having a higher average TP than the consumer, some of the known predator-prey interactions prey had higher TP than the assumed predator due to the prey's higher $\delta^{15}N$ signal. For example, both northern pike and the great cormorant had higher TP than the white-tailed eagle, despite white-tailed eagle being the avian apex species in the Baltic Sea food web. However, large proportion of the white-tailed eagle diet consists of lower TP species, such as ducks, including the common eider, which has been found to be most consumed prey species in Finnish coast [4, 5]. The white-tailed eagle positioned below many of the fish species and also the piscivorous great cormorant, but above the common eider, which would be expected if the eagles included in our study consumed a large proportion of eiders or other ducks instead of fish or

piscivorous birds. White-tailed eagles may also feed from terrestrial food webs, where they typically prey on herbivores, and that will lower their $\delta^{15}N$ signal.

4. Supplementary references

[1] Cabana G, Rasmussen J (1996) Comparison of aquatic food chains using nitrogen isotopes. *Proceedings of the National Academy of Science of the United States of America* 93: 10844-10847

[2] Post DM (2002) Using stable isotopes to estimate trophic position: Models, methods, and assumptions. *Ecology* 83:703–718.

[3] Beldowska M, Kobos J (2018) The variability of Hg concentration and composition of marine phytoplankton. *Environmental Science and Pollution and Research*

[4] Sulkava S, Tornberg R, Koivusaari J (1997) Diet of the White-tailed Eagle *Haliaeetus albicilla* in Finland. *Ornis Fennica* 74:65–78

[5] Ekblad CMS, Sulkava S, Stjernberg TG, Laaksonen TK (2016) Landscape-Scale Gradients and Temporal Changes in the Prey Species of the White-Tailed Eagle (*Haliaeetus albicilla*). *Annales Zoologici Fennici* 53:228–240. doi: 10.5735/086.053.0401

5. Equations

$$BMF_{TP} = \frac{[Hg_{predator}]/[Hg_{prey}]}{TP_{predator} - TP_{prey}} \quad (1)$$

$$BMF_{R} = [Hg_{predator}]/[Hg_{prey}] \quad (2)$$

6. References to support food chain classification

Froese R, Pauly D (eds) (2021) FishBase. www.fishbase.org.

Gagnon K, Gräfnings M, Boström C (2019). Trophic role of the mesopredatory three-spined stickleback in habitats of varying complexity. *Journal of Experimental Marine Biology and Ecology* 510: 46–53. https://doi.org/10.1016/j.jembe.2018.10.003

Gutsch M, Hoffman J (2016) A review of Ruffe (*Gymnocephalus cernua*) life history in its native versus non-native range. *Reviews in Fish Biology and Fisheries* 26: 213–233. https://doi.org/10.1007/s11160-016-9422-5

Hägerstrand H, Himberg M, Saks L, Verliin A (2018). Stomach content of whitefish, *Coregonus lavaretus* (Actinopterygisalmoniformes: Salmonidae), off the Åland islands, Baltic Sea. *Acta Ichthyologica et Piscatoria* 48: 71–74. https://doi.org/10.3750/AIEP/02260

Herlevi H, Aarnio K, Puntila-Dodd R, Bonsdorff E (2018). The food web positioning and trophic niche of the non-indigenous round goby: a comparison between two Baltic Sea populations. *Hydrobiologia* 822: 111–128. https://doi.org/10.1007/s10750-018-3667-z

Jacobson P, Bergström U, Eklöf J (2019). Size-dependent diet composition and feeding of Eurasian perch (*Perca fluviatilis*) and northern pike (*Esox lucius*) in the Baltic Sea. *Boreal Environment Research* 24: 137–153.

Lankov A, Ojaveer H, Simm M, Põllupüü M, Möllmann C (2010). Feeding ecology of pelagic fish species in the Gulf of Riga (Baltic Sea): The importance of changes in the zooplankton community. *Journal of Fish Biology* 77: 2268–2284. https://doi.org/10.1111/j.1095-8649.2010.02805.x

Lappalainen A, Westerbom M, Heikinheimo O (2005). Roach (*Rutilus rutilus*) as an important predator on blue mussel (*Mytilus edulis*) populations in a brackish water environment, the northern Baltic Sea. *Marine Biology* 147: 323–330. https://doi.org/10.1007/s00227-005-1598-5

Mustamäki N, Cederberg T, Mattila J (2014). Diet, stable isotopes and morphology of Eurasian perch (*Perca fluviatilis*) in littoral and pelagic habitats in the northern Baltic Proper. *Environmental Biology of Fishes* 97: 675–689. https://doi.org/10.1007/s10641-013-0169-8

Puntila-Dodd R, Loisa O, Riipinen K, Fowler AE (2019). A taste for aliens: contribution of a novel prey item to native fishes' diet. *Biological Invasions* 21: 2907–2917. https://doi.org/10.1007/s10530-019-02021-w

Taal I, Saks L, Nedolgova S, Verliin A, Kesler M, Jürgens K, Svirgsden R, Vetemaa M, Saat, T. (2014). Diet composition of smelt Osmerus eperlanus (Linnaeus) in brackish near-shore ecosystem (Eru Bay, Baltic Sea). *Ecology of Freshwater Fish* 23: 121–128. https://doi.org/10.1111/eff.12044

Van Deurs, M., Christensen, A., & Rindorf, A. (2013) Patchy zooplankton grazing and high energy conversion efficiency: ecological implications of sandeel behavior and strategy. *Marine Ecology Progress Series* 487, 123–133. https://doi.org/10.2307/24892114

Verliin A, Kotta J, Orav-Kotta H, Saks L, Vetemaa M (2011). Food selection of Coregonus lavaretus in a brackish water ecosystem. *Journal of Fish Biology* 78: 540–551. https://doi.org/10.1111/j.1095-8649.2010.02870.x

Westerbom M, Lappalainen A, Mustonen O, Norkko A (2018). Trophic overlap between expanding and contracting fish predators in a range margin undergoing change. *Scientific Reports* 8: 7895. https://doi.org/10.1038/s41598-018-25745-6

Table S1. List of study species, sample size (*n*), sampling time, location and method, and analysed tissues. *: composite sample.

Scientific name	Common name	*n*	Food chain	Sampling time	Sampling method	Tissue
**Birds**						
Haliaeetus albicilla	white-tailed eagle	10	benthopelagic	2013–2019	carcasses (Finnish Natural History Museum)	muscle + liver
Phalacrocorax carbo	great cormorant	6	benthopelagic	08/2018	shot by hunters	muscle + liver
Somateria mollissima	common eider	13	benthic	08/2018	shot by hunters	muscle + liver
**Fish**						
Abramis brama	common bream	6	benthic	2017	fishing net	muscle
Ammodytes tobianus	lesser sand eel	1*	pelagic	05/2019	drag net	whole
Clupea harengus	Baltic herring	10	pelagic	11-12/2017	fishing net	muscle
Coregonus albula	vendace	2	pelagic	11/2017	bottom trawl	whole
Coregonus lavaretus	common whitefish	2	benthopelagic	05/2019	fishing net	muscle
Esox lucius	northern pike	9	benthopelagic	11-12/2017–06/2018	fishing net + fish market	muscle
Gasterosteus aculeatus	three-spined stickleback	6*	benthopelagic	05/2019	drag net	whole
Gobius niger	black goby	5	benthic	05/2019	trap cage	whole
Gymnocephalus cernua	Eurasian ruffe	10	benthic	05/2019	fishing net	muscle
Myoxocephalus quadricornis	fourhorn sculpin	5	benthic	11/2017	fishing net	muscle
Neogobius melanostomus	round goby	10	benthic	05/2019	fishing net + trap cage	whole
Osmerus eperlanus	European smelt	10	benthopelagic	11/2017	fishing net	whole
Perca fluviatilis	European perch	10	benthopelagic	10-12/2017	fishing net	muscle
Pomatoschistus minutus	sand goby	1*	benthic	05/2019	drag net	whole
Rutilus rutilus	common roach	10	benthic	10-11/2017	fishing net	muscle
Salmo salar	Atlantic salmon	1	pelagic	06/2019	fish market	muscle
Zoarces viviparus	viviparous eelpout	10	benthic	11/2017	fishing net + bottom trawl	whole
**Invertebrates**						
Gammarus spp.		6*	benthic	10-11/2017	by hand	whole
Idotea spp.		4*	benthic	10-11/2017	by hand	whole
Macoma balthica	Baltic clam	4*	benthic	10/2017	sediment sampler + hand-picking	soft tissue
Mytilus edulis/trossulus	blue mussel	9*	benthic	10-11/2017–06-07/2018	by hand	soft tissue
Palaemon adspersus		1	benthic	05/2019	drag net	whole
Palaemon elegans		1*	benthic	05/2019	drag net	whole
Rhithropanopeus harrisii	Harris mud crab	1*	benthic	05/2019	trap cage	soft tissue
Saduria entomon		2*	benthic	11/2017	bottom trawl	whole
Theodoxus fluviatilis		3*	benthic	10-11/2017	by hand	whole
**Plankton**						
phytoplankton	phytoplankton	3*	pelagic	05/2019	20–50 µm net	whole
zooplankton	zooplankton	2*	pelagic	05/2019	100 µm net	whole
**Primary producers**						
Cladophora glomerata		3*	benthic	05/2019	hand-picking	whole
Fucus spp.	bladderwrack	3*	benthic	09-11/2017	hand-picking	whole
Pilayella/Ectocarpus spp.		3*	benthic	05/2019	hand-picking	whole

Table S2. Body measurements of the study species. Sample sizes (*n*) marked with *: composite sample. Empty cells indicate information is not available.

		Length (cm)			Body mass (g)		
	n	**Mean ± *SD***	**median**	**Min. – max.**	**Mean ± *SD***	**Median**	**Min. – max.**
**Birds**							
Haliaeetus albicilla	7				4840.0 ± 1033.4	4900.0	3300–6000
Phalacrocorax carbo	6						
Somateria mollissima	13						
**Fish**							
Abramis brama	6	25.9 ± 4.5	24.0	22.6–31	507.0 ± 319.0	515.0	170–950
Ammodytes tobianus	1*			5.1–7.4	0.6 ± 0.2	0.6	0.3–1.2
Coregonus albula	2	12.0 ± 1.4		11–13	7.5 ± 3.2	7.5	5.2–9.7
Coregonus lavaretus	2	20.4 ± 0.1		20.3–20.5	23.8 ± 2.0	23.8	22.4–25.2
Clupea harengus	10	15.0 ± 1.9	15.2	11.4–18	14.6 ± 14.8	6.5	5.1–44.2
Esox lucius	9				>1000		
Gasteosterus aculeatus	6	5.9 ± 0.6	6.0	5–6.4	2.0 ± 0.2	1.9	1.8–2.3
Gobius niger	5	8.2 ± 0.5	8.0	7.7–9.0	7.3 ± 1.2	6.9	5.7–8.8
Gymnocephalus cernua	10	13.7 ± 1.1	13.7	12.3–15.2	6.5 ± 1.1	6.7	4.8–8.7
Myoxocephalus quadricornis	5	24.0 ± 3.3	23.5	19.8–29	9.7 ± 2.8	10.9	6.6–12.2
Neogobius melanostomus	10	12.5 ± 1.5	11.8	11–15.3	31.2 ± 12.8	29.4	17.5–53.5
Osmerus eperlanus	10	11.9 ± 1.4	11.5	9.7–14.5	7.7 ± 2.9	6.8	3.3–12.8
Perca fluviatilis	10	21.9 ± 5.3	23.2	14–29.8	15.2 ± 4.4	15.0	8.4–21.6
Pomatoschistus minutus	1*	4.6 ± 0.4	4.6	4.2–5.5	0.6 ± 0.1	0.6	0.4–0.9
Rutilus rutilus	10	15.7 ± 2.5	15.1	12.5–19.9	17.0 ± 16.2	10.4	5.3–59.2
Salmo salar	1						
Zoarces viviparus	10	14.2 ± 3.2	13.4	10.8–22	12.0 ± 11.1	8.3	4.3–41.4
**Invertebrates**							
Gammarus spp.	6*			0.9–1.9			
Idotea spp.	4*			1.2–2.1			
Macoma balthica	4*			0.7–2.0			
Mytilus edulis/trossulus	9			0.76–3.4			
Palaemon elegans	1*			1.6–3.0			
Palaemon adspersus	1			3.2			
Rhitropanopeus harrisii	1*			0.6–1.6			
Saduria entomon	2*			2.3–5.0			
Theodoxus fluviatilis	3*			0.4–0.8			

Table S3. Summary statistics for $\delta^{15}N$, C:N ratio, dry and wet weight Hg concentrations, and dry matter content for the study species.

		C:N		δ15N (‰)		dry matter		Hg (*dw, µg g*-1)		Hg (*ww, µg g*-1)	
	n	**mean ± *SD***	**median (range)**	**mean ± SD**	**median (range)**	**mean ± *SD***	**median (range)**	**mean ± *SD***	**median (range)**	**mean ± SD**	**median (range)**
**Birds**											
Haliaeetus albicilla	7	4.32 ± 0.6	4.42 (3.38 - 5.06)	+12.09 ± 1.83	+11.71 (+9.07 - +15.72)	0.30 ± 0.04	0.31 (0.22 - 0.35)	1.86 ± 1.66	1.35 (0.53 - 6.31)	0.506 ± 0.342	0.421 (0.176 - 1.403)
Phalacrocorax carbo	6	3.94 ± 0.4	3.83 (3.58 - 4.56)	+14.48 ± 1.86	+14.87 (+11.09 -+16.08)	0.28 ± 0.02	0.27 (0.25 - 0.32)	1.28 ± 1.01	0.91 (0.53 - 3.28)	0.350 ± 0.270	0.253 (0.138 - 0.885)
Somateria mollissima	13	3.60 ± 0.2	3.56 (3.17 - 4.97)	+10.55 ± 0.79	+10.33 (+9.26 - +12.05)	0.26 ± 0.04	0.28 (0.18 - 0.30)	0.67 ± 0.31	0.59 (0.26 - 1.38)	0.147 ± 0.089	0.144 (0.071 - 0.388)
**Fish**											
Abramis brama	6	3.32 ± 0.17	3.25 (3.16 - 3.58)	+13.80 ± 1.37	+13.54 (+12.23 - +16.02)	0.25 ± 0.04	0.24 (0.21 - 0.31)	0.17 ± 0.06	0.18 (0.10 - 0.25)	0.000 ± 0.000	0.041 (0.023 - 0.068)
Ammodytes tobianus	1*	3.81	3.81	+10.73	+10.73	0.20	0.20	0.01	0.01	0.003	0.003
Coregonus albula	2	3.36 ± 0.01	3.36 (3.35 - 3.36)	+12.30 ± 0.22	+12.29 (+12.14 - +12.45)	0.21 ± 0.001	0.21 (0.21 - 0.21)	0.09 ± 0.01	0.09 (0.08 - 0.10)	0.02 ± 0.003	0.018 (0.016 - 0.020)
Coregonus lavaretus	2	3.29 ± 0.04	3.29 (3.26 - 3.32)	+11.85 ± 0.16	+11.85 (+11.74 - +11.97)	0.23 ± 0.01	0.23 (0.23 - 0.24)	0.08 ± 0.05	0.08 (0.05 - 0.12)	0.02 ± 0.01	0.019 (0.012 - 0.027)
Clupea harengus	10	3.68 ± 0.28	3.69 (3.35 - 4.25)	+13.00 ± 2.39	+12.38 (+10.25 - +18.01)	0.23 ± 0.02	0.23 (0.20 - 0.25)	0.15 ± 0.17	0.10 (0.03 - 0.61)	0.03 ± 0.05	0.023 (0.005 - 0.146)
Esox lucius	9	3.10 ± 0.04	3.10 (3.06 - 3.16)	+15.84 ± 0.46	+15.72 (+15.17 - +16.60)	0.22 ± 0.01	0.21 (0.20 - 0.24)	0.68 ± 0.43	0.54 (0.19 - 1.53)	0.05 ± 0.09	0.112 (0.044 - 0.337)
Gasterosteus aculeatus	6	4.43 ± 0.57	4.38 (3.67 - 5.31)	+11.52 ± 0.47	+11.55 (+10.76 - +12.06)	0.27 ± 0.03	0.26 (0.24 - 0.32)	0.04 ± 0.02	0.05 (0.01 - 0.05)	0.01 ± 0.004	0.012 (0.004 - 0.014)
Gobius niger	5	3.66 ± 0.15	3.61 (3.56 - 3.93)	+11.80 ± 0.27	+11.66 (+11.58 - +12.21)	0.21 ± 0.01	0.21 (0.21 - 0.23)	0.03 ± 0.01	0.03 (0.02 - 0.04)	0.01 ± 0.002	0.007 (0.004 - 0.008)
Gymnocephalus cernua	10	3.43 ± 0.15	3.38 (3.32 - 3.84)	+14.40 ± 0.69	+14.62 (+13.38 - +15.30)	0.21 ± 0.01	0.21 (0.19 - 0.23)	0.12 ± 0.08	0.09 (0.05 - 0.27)	0.03 ± 0.02	0.019 (0.011 - 0.053)
Myoxocephalus quadricornis	5	3.30 ± 0.08	3.29 (3.21 - 3.39)	+16.59 ± 0.75	+16.23 (+16.97 - +17.68)	0.20 ± 0.03	0.21 (0.16 - 0.22)	0.45 ± 0.39	0.31 (0.16 - 1.12)	0 ± 0	0.065 (0.032 – 0.176)
Neogobius melanostomus	10	4.02 ± 0.27	4.06 (3.71 - 4.44)	+10.45 ± 0.64	+10.43 (+9.48 - +11.55)	0.24 ± 0.01	0.25 (0.23 - 0.25)	0.02 ± 0.01	0.02 (0.01 - 0.04)	0.004 ± 0.002	0.004 (0.002 - 0.009)
Osmerus eperlanus	10	3.41 ± 0.09	3.39 (3.31 - 3.55)	+12.78 ± 0.65	+12.66 (+11.82 - +13.77)	0.21 ± 0.02	0.21 (0.19 - 0.23)	0.12 ± 0.09	0.11 (0.02 - 0.29)	0.02 ± 0.02	0.023 (0.004 - 0.059)
Perca fluviatilis	10	3.27 ± 0.13	3.24 (3.13 - 3.59)	+13.73 ± 1.68	+14.00 (+10.30 - +15.67)	0.20 ± 0.02	0.21 (0.18 - 0.23)	0.48 ± 0.38	0.32 (0.12 - 1.14)	0.01 ± 0.02	0.064 (0.027 - 0.211)
Pomatoschistus minutus	1*	3.70	3.70	+13.73	+13.73	0.24	0.24	0.02	0.019	0.005	0.005
Rutilus rutilus	10	3.31 ± 0.07	3.31 (3.23 - 3.42)	+11.67 ± 1.58	+11.72 (+8.86 - +14.86)	0.23 ± 0.01	0.22 (0.22 - 0.26)	0.15 ± 0.06	0.14 (0.08 - 0.25)	0.04 ± 0.01	0.031 (0.018 - 0.056)
Salmo salar	1	3.64	3.64	+13.00	+13.00	0.37 ± 0	0.37	0.15	0.15	0.05	0.055
Zoarces viviparus	10	3.91 ± 0.22	3.96 (3.37 - 4.11)	+12.78 ± 0.49	+12.70 (+12.08 - +13.61)	0.24 ± 0.02	0.24 (0.20 - 0.26)	0.07 ± 0.04	0.06 (0.02 - 0.12)	0.02 ± 0.01	0.015 (0.006 - 0.028)
**Invertebrates**											
Gammarus spp.	6*	4.97 ± 0.17	4.91 (4.74 - 5.17)	+4.15 ± 0.84	+4.15 (+2.85 - +5.36)	0.21 ± 0.02	0.22 (0.19 - 0.23)	0.02 ± 0.023	0.009 (0.001 - 0.07)	0.004 ± 0.005	0.002 (0.000 - 0.014)
Idotea spp.	4*	6.68 ± 0.82	6.71 (5.69 - 7.59)	+4.25 ± 1.22	+4.10 (+3.14 - +5.65)	0.25 ± 0.04	0.26 (0.20 - 0.30)	0.006 ± 0.004	0.005 (0.003 - 0.011)	0.002 ± 0.001	0.001 (0.001 - 0.003)
Macoma balthica	4*	5.92 ± 0.25	5.92 (5.63 - 6.22)	+8.70 ± 0.75	+8.60 (+8.46 - +9.13)	0.2 ± 0.01	0.20 (0.19 - 0.21)	0.04 ± 0.006	0.04 (0.03 - 0.04)	0.007 ± 0.001	0.008 (0.006 - 0.009)
Mytilus edulis/trossulus	9	5.00 ± 0.53	5.19 (4.23 - 5.51)	+7.16 ± 1.40	+7.67 (+5.57 - +9.59)	0.16 ± 0.02	0.16 (0.13 - 0.19)	0.04 ± 0.01	0.04 (0.01 - 0.05)	0.006 ± 0.002	0.006 (0.002 - 0.009)
Palaemon adpersus	1	3.61	3.61	+9.90	+9.90	0.23	0.23	0.009	0.009	0.002	0.002
Palaemon elegans	1*	3.86	3.86	+8.44	+8.44	0.25	0.25	0.009	0.009	0.002	0.002
Rhitropanopeus harrisii	1*	6.36	6.36	+8.24	+8.24	0.25	0.25	0.03	0.03	0.007	0.007
Saduria entomon	2*	5.58 ± 0.87	5.75 (5.14 - 6.37)	+10.55 ± 0.79	+10.55 (+9.99 - +11.10)	0.31 ± 0.10	0.31 (0.25 - 0.38)	0.05 ± 0.03	0.05 (0.03 - 0.07)	0.02 ± 0.01	0.016 (0.006 - 0.026)
Theodoxus fluviatilis	3*	8.85 ± 2.46	9.25 (6.21 - 11.09)	+4.29 ± 0.53	+4.26 (+3.77 - +4.83)	0.52 ± 0.10	0.49 (0.44 - 0.64)	0.005 ± 0.005	0.004 (0 - 0.01)	0.002 ± 0.002	0.002 (0.000 - 0.004)
**Plankton**											
phytoplankton	2*	8.57 ± 1.32	8.56 (7.6- 9.50)	+6.25 ± 1.08	+6.25 (+5.49 - +7.01)	0.18 ± 0.09	0.18 (0.12 - 0.25)	0.14 ± 0.14	0.14 (0.04 - 0.24)	0.02 ± 0.01	0.020 (0.011 - 0.029)
zooplankton	3*	5.87 ± 0.17	5.84 (5.7 - 6.05)	+7.52 ± 0.14	+7.52 (+7.38 - +7.65)	0.10 ± 0.02	0.09 (0.09 - 0.11)	0.006 ± 0.002	0.007 (0.004 - 0.007)	0.001 ± 0	0.001 (0.000 - 0.001)
**Primary producers**											
Fucus spp.	3*	48.74 ± 27.67	64.66 (16.78 - 64.76)	+4.64 ± 1.92	+3.83 (+3.26 - +6.84)	0.21 ± 0.02	0.20 (0.19 - 0.23)	0.003 ± 0.003	0.002 (0.002 - 0.007)	0.001 ± 0.001	0.000 (0.000 - 0.001)
Cladophora glomerata	3*	19.44 ± 2.51	20.46 (16.58 - 21.28)	+4.79 ± 0.73	+4.73 (+4.09 - +5.55)	0.17 ± 0.08	0.19 (0.08 - 0.24)	0.002 ± 0.002	0.002 (0 - 0.005)	0 ± 0	0.000 (0.000- 0.001)
Pilayella spp.	3*	22.90 ± 3.25	23.85 (19.28 - 25.58)	+4.99 ± 0.12	+5.02 (+4.87 - +5.10)	0.17 ± 0.04	0.18 (0.12 - 0.21)	0.007 ± 0.002	0.008 (0.005 - 0.008)	0.001 ± 0	0.001 (0.001 - 0.002)

Table S4. Biomagnification factors (BMFs) for benthic predator-prey pairs using median, minimum, and maximum dry weight Hg concentrations (per Eq. 1).

Predator / Prey	*Gammarus spp.*	*Idotea spp.*	*Theodoxus fluviatilis*	*Palaemon adpersus*	*Palaemon elegans*	*Saduria entomon*	*Rhithropanopeus harrisii*	*Coregonus lavaretus*	*Abramis brama*	*Rutilus rutilus*	*Gymnocephalus cernua*	*Osmerus eperlanus*
Cladophora glomerata												
Pilayella/Ectocarpus spp.												
Fucus spp.												
Gammarus spp.						2.9 (9.8 -0.5)	2.7 (17.7 - 0.3)	4.3 (16.3 - 0.7)	7.2 (25.5 - 1.3)	7.3 (27.7 - 1.6)	3.4 (12.3 - 1.3)	5.1 (5.7 - 1.6)
Idotea spp.						4.9 (4.7 - 3.4)	4.7 (8.5 - 2.2)	7.3 (7.8 - 4.8)	12.1 (12.1 - 8.1)	12.3 (13.2 -10.5)	5.8 (5.8 - 8.2)	8.6 (2.7 - 10.4)
Theodoxus fluviatilis						6.5 (67.9 - 3.7)	6.2 (123.4 - 2.4)	9.6 (111.8 - 5.2)	15.9 (174.6 - 8.8)	16.1 (190.6 - 11.4)	7.6 (84.0 - 8.9)	11.3 (39.4 - 11.3)
Palaemon adpersus						28.6 (15 - 42.3)		17 (9.9 - 24.1)	17.8 (9.8 - 25.5)	30.9 (18.2 - 56.5)	7.8 (4.3 - 23.8)	15.3 (2.7 - 39.5)
Palaemon elegans						8.3 (4.4 - 12.3)		9.2 (5.4 - 13)	12.3 (6.7 - 17.5)	16 (9.4 - 29.3)	5.6 (3.1 -16.9)	9.6 (1.7 - 24.8)
Saduria entomon								4.7 (5.2 - 4.5)	3.9 (4.1 - 3.8)	8.9 (10.0 - 11.0)	1.7 (1.8 - 3.4)	
Rhithopanopeus harrisii								2.8 (1.6 - 4)	3.8 (2.1 - 5.4)	4.9 (2.9 - 8.9)	1.7 (1.0 - 5.3)	
Mytilus edulis/trossulus						1.2 (2.4 - 1.3)	2.3 (8.7 - 1.7)	1.6 (3.5 - 1.6)	2.4 (4.8 - 2.4)	2.8 (6.0 - 3.6)	1.1 (2.2. - 2.4)	
Macoma balthica						2.2 (1.6 - 3.0)		2.3 (1.9 - 3.0)	3.0 (2.3 - 3.9)	4.1 (3.3 - 6.8)	1.4 (1.0 - 3.8)	

Table S4. Continued.

Prey \ Predator	*Gasterosteus aculeatus*	*Zoarces viviparus*	*Gobius niger*	*Neogobius melanostomus*	*Pomatoschistus minutus*	*Myoxocephalus quadricornis*	*Perca fluviatilis*	*Esox lucius*	*Somateria mollissima*	*Phalacrocorax carbo*	*Haliaeetus albicilla*
Gammarus spp.	2.6 (4.7 - 0.4)	2.8 (6.8 - 0.7)	1.7 (5.7 - 0.2)	1.0 (4.1 - 0.3)	0.8 (5.1 -0.1)	9.8 (32.9 - 4.4)	13.5 (33.2 -5.7)				
Idotea spp.	4.4 (2.2 -2.3)	4.7 (3.3 - 4.4)	2.8 (2.7 - 1.5)	1.7 (1.9 - 1.8)	1.3 (2.4 - 0.6)	16.3 (15.6 - 28.0)	22.5 (15.8 - 37.1)				
Theodoxus fluviatilis	5.7 (32.3 - 2.5)	6.2 (47.0 - 4.8)	3.7 (39.1 - 1.6)	2.3 (28.0 - 2.0)	1.7 (34.6 - 0.7)	21.4 (224.7 -30.5)	29.6 (227.4 - 40.3)				
Palaemon adpersus	11.8 (3.3 - 13.3)	8.3 (3.2 - 16.6)	6.7 (3.5 -7.7)	11.5 (7.1 - 26.4)		18.1 (9.5 - 66.6)	33.5 (12.8 - 118.2)				
Palaemon elegans	5.8 (1.6 - 6.6)	5.2 (2.0 - 10.4)	3.6 (1.9 - 4.1)	3 (1.9 - 6.9)		14 (7.3 - 51.6)	22.9 (8.8 - 80.9)				
Saduria entomon		2 (1.4 - 2.7)	1.9 (1.9 - 1.5)			3.7 (3.7 - 9.1)	7.4 (5.4 - 17.6)				
Rhitopanopeus harrisii		1.6 (0.6 - 3.2)	1.1 (0.6 - 1.3)	0.9 (0.5 - 2.0)		4.4 (2.3 - 16.2)	7.1 (2.7 - 25.1)				
Mytilus edulis/trossulus		1.0 (1.4. -1.4)	0.6 (1.2 - 0.5)	0.4 (1.0 - 0.7)		2.9 (5.6 - 7.7)	4.4 (6.3 - 11.3)		12.8 (20.9 - 21.5)		
Macoma balthica		1.3 (0.7 -2.4)	0.9 (0.7 - 1.0)	0.8 (0.7 - 1.7)		3.4 (2.4 - 11.3)	5.7 (2.9 - 18.1)		19.9 (12 - 42)		
Coregonus lavaretus						2.6 (2.3 - 6.8)	7 (4.6 -17.4)	5.5 (3.4 - 11)		10.2 (10.1 - 25.9)	44.3 (29.6 - 145.8)
Abramis brama								5.1 (3.4 -10.2)		10.5 (11.1 - 26.5)	
Rutilus rutilus						1.5 (1.4 - 3.1)	3.9 (2.5 - 7.5)	3.2 (2.0 - 4.9)		5.9 (5.8 - 11.6)	23.4 (15.5 - 59.9)
Gymnocephalus cernua						5.3 (5.0 - 6.5)		14.3 (9.3 - 13.3)		32.3 (33.7 - 38.2)	
Osmerus eperlanus						2.5 (7.4 - 3.5)	10.4 (22.9 - 14.2)	5.4 (11.2 - 5.9)		10.4 (34.3 -14.4)	133.6 (299.1 - 240.8)
Gasterosteus aculeatus						4.3 (7.9 - 13.9)	10.3 (14.1 - 32.3)	9.8 (9.4 -14.0)			
Zoarces viviparus						4.5 (6.2 - 8.3)	19.2 (19.3 - 33.8)	9.9 (9.4 - 14)		19 (28.8 - 34.2)	246.8 (253.0 - 576.4)
Gobius niger						6.7 (6.7 - 21.5)	17.6 (12.9 - 54.3)	14 (9.7 - 34.7)		26.1 (28.7 -81.9)	
Neogobius melanostomus						10.5 (8.9 - 16.9)	20.8 (12.9 - 32.1)	21 (12.3 - 26.2)		38.4 (35.7 - 60.4)	
Pomatoschistus minutus						19.2 (10.0 - 70.7)					
Myoxocephalus quadricornis											
Perca fluviatilis						1.1 (1.5 - 1.2)		2.7 (2.5 -2.2)		5.5 (8.3 - 5.6)	
Esox lucius											
Somateria mollissima											4.4 (3.9 - 8.8)
Phalacrocorax carbo											

Table S5. Biomagnification factors (BMFs) for pelagic predator-prey pairs using median, minimum, and maximum dry weight Hg concentrations (per Eq. 1).

Predator / Prey	*zoo-plankton*	*Clupea harengus*	*Salmo salar*	*Coregonus lavaretus*	*Osmerus eperlanus*	*Perca fluviatilis*	*Ammodytes tobianus*	*Coregonus albula*	*Gasterosteus aculeatus*	*Esox lucius*	*Phalacrocorax carbo*	*Haliaeetus albicilla*
phytoplankton												
zooplankton		9.0 (3.7 - 54.7)		9.9 (9.3 - 13.5)	10.8 (3.0 - 26.9)		2.2 (3.6 - 2.1)	9.3 (13.4 - 10.0)	6.2 (2.8 -6.7)			
Clupea harengus			1421.8 (5503.8 -223.7)			15.7 (23.2 - 8.7)				6.7 (9.4 - 3.0)	13.1 (29.2 - 7.4)	508.4 (768.2 - 373.4)
Salmo salar												338.5 (132.1 - 1580.2)
Coregonus lavaretus			5.3 (9.1 - 3.7)			7.0 (4.6 - 17.4)				5.5 (3.4 - 11.0)	10.2 (10.1 - 32.3)	44.3 (29.6 - 145.8)
Osmerus eperlanus			20.9 (119.8 - 8.1)			10.4 (22.9 - 14.2)				5.4 (11.2 - 5.9)	10.4 (34.3 - 14.4)	133.6 (299.1 - 240.8)
Perca fluviatilis										2.7 (2.5 - 2.2)	5.5 (8.3 - 5.6)	
Ammodytes tobianus						26.2, 92.5, 10.0						
Coregonus albula			8.3 (9.3 - 7.5)			8.8 (3.8 - 28.1)				5.9 (2.4 - 15.2)	11.2 (7.2 -36.4)	66.5 (29.1 - 280.2)
Gasterosteus aculeatus			7.1 (25.4 - 6.3)			10.3 (14.1 - 32.3)						
Esox lucius												
Phalacrocorax carbo												

Table S6. Biomagnification factors (BMFs) for benthic predator-prey pairs using median, minimum, and maximum dry weight Hg concentrations (per Eq. 2).

Prey \ Predator	***Gammarus* spp.**	***Idotea* spp.**	***Theodoxus fluviatilis***	***Palaemon adpersus***	***Palaemon elegans***	***Saduria entomon***	***Rhithropanopeus harrisii***	***Coregonus lavaretus***	***Abramis brama***	***Rutilus rutilus***	***Gymnocephalus cernua***	***Osmerus eperlanus***
Cladophora glomerata	5.4 (5.9 - 14.3)	3.3 (12.5 - 2.2)	2.5 (0.9 - 2.1)	5.4 (37.9 - 1.7)	5.7 (40.1 - 1.8)							
***Pilayella/ Ectocarpus* spp.**	1 (0.3 - 8.4)	0.6 (0.6 - 1.3)	0.5 (0.4 - 1.2)	1.0 (1.9 - 1.0)	1.1 (2.0 - 1.1)							
***Fucus* spp.**	4.1 (0.8 - 10.8)	2.5, (1.8 -1.7)	1.9 (0.1 - 1.6)	4.2 (5.3 - 1.3)	4.4 (5.7 - 1.4)							
***Gammarus* spp.**						5.5 (18.5 - 1.0)	3.3 (21.2 - 0.4)	9.8 (36.8 - 1.7)	20.6 (72.3 - 3.5)	16.2 (61.2 - 3.6)	10.4 (36.9 - 3.8)	13 (14.6 - 4.1)
***Idotea* spp.**						9.1 (8.7 - 6.3)	5.5 (10.0 - 2.5)	16.3 (17.4 - 10.8)	34 (34.1 - 22.7)	26.8 (28.9 - 22.8)	17.2 (17.4 - 24.4)	21.5 (6.9 - 26.0)
Theodoxus fluviatilis						11.9 (125.1 - 6.8)	7.2 (143.4 - 2.8)	21.3 (248.8 - 11.7)	44.6 (488.6 - 24.6)	35 (413.8 - 24.8)	22.5 (249.7 - 26.4)	28.2 (98.5 -28.2)
Palaemon adpersus						5.5 (2.9 - 8.1)	3.3 (3.3 - 3.3)	9.8 (5.7 - 13.9)	20.5 (11.2 - 29.2)	16.1 (9.5 - 29.4)	10.3 (5.7 - 31.4)	12.9 (2.3 - 33.5)
Palaemon elegans						5.2 (2.7 - 7.6)	3.1 (3.1 - 3.1)	9.2 (5.4 - 13.1)	19.3 (10.6 - 27.6)	15.2 (9.0 - 27.8)	9.8 (5.4 - 29.7)	12.2 (2.1 - 31.7)
Saduria entomon								1.8 (2.0 - 1.7)	3.7 (3.9 - 3.6)	2.9 (3.3 - 3.6)	1.9 (2.0 - 3.9)	
Rhithopanopeus harrisii								3 (1.7 - 4.2)	6.2 (3.4 - 8.9)	4.9 (2.9 - 9.0)	3.1 (1.7- 9.6)	
Mytilus edulis/trossulus						1.2 (2.4 - 1.3)	0.7 (2.7 - 0.5)	2.2 (4.8 - 2.3)	4.6 (9.3 - 4.8)	3.6 (7.9 - 4.8)	2.3 (4.8 - 5.1)	
Macoma balthica						1.2 (0.9 - 1.6)	0.7 (1.0 - 0.7)	2.2 (1.7 - 2.8)	4.6 (3.4 - 5.9)	3.6 (2.9 - 5.9)	2.3 (1.7 - 6.3)	

Table S6. Continued.

Predator / Prey	*Gasterosteus aculeatus*	*Zoarces viviparus*	*Gobius niger*	*Neogobius melanostomus*	*Pomaschistus minutus*	*Myoxocephalus quadricornis*	*Perca fluviatilis*	*Esox Lucius*	*Somateria mollissima*	*Phalacrocorax carbo*	*Haliaeetus albicilla*
Gammarus spp.	5.6 (10.2 - 0.8)	7.1 (17.4 - 1.7)	3.8 (12.8 - 0.5)	1.9 (7.5 - 0.5)	2.2 (14.2 -0.3)	35.8 (120.3 - 15.9)	37.9 (93.5 - 16.2)				
Idotea spp.	9.3 (4.8 - 4.9)	11.7 (8.2 - 11.0)	6.3 (6.0 - 3.4)	3.1 (3.5 - 3.3)	3.7 (6.7 - 1.7)	59.3 (56.7 - 101.7)	62.8 (44.1 - 103.4)				
Theodoxus fluviatilis	12.2 (68.7 - 5.3)	15.4 (117.4 - 11.9)	8.2 (86.5 -3.6)	4.1 (50.8 - 3.6)	4.8 (96.2 - 1.9)	77.6 (813.3 - 110.3)	82.2 (631.7 - 112.1)				
Palaemon adpersus	5.6 (1.6 - 6.3)	7.1 (2.7 - 14.1)	3.8 (2.0 - 4.3)	1.9 (1.2 - 4.3)		35.7 (18.6 - 131.1)	37.8 (14.5 - 133.3)				
Palaemon elegans	5.3 (1.5 - 6)	6.7 (2.5 - 13.3)	3.6 (1.9 - 4.1)	1.8 (1.1 - 4.1)		33.7 (17.6 -123.9)	35.7 (13.7 - 125.9)				
Saduria entomon		1.3 (0.9 - 1.7)	0.7 (0.7 - 0.5)	0.3 (0.4 - 0.5)		6.5 (6.5 - 16.2)	6.9 (5.1 - 16.5)				
Rhitopanopeus harrisii		2.2 (0.8 - 4.3)	1.2 (0.6 - 1.3)	0.6 (0.4 - 1.3)		10.9 (5.7 - 39.9)	11.5 (4.4 - 40.6)				
Mytilus edulis/trossulus		1.6 (2.2 - 2.3)	0.9 (1.7 - 0.7)	0.4 (1.0 - 0.7)		8.1 (15.6 - 21.4)	8.6 (12.1 - 21.8)		15.7 (25.6 - 26.4)		
Macoma balthica		1.6 (0.8 - 2.8)	0.8 (0.6 - 0.9)	0.4 (0.4 - 0.9)		7.9 (5.6 - 26.3)	8.4 (4.4 - 26.8)		15.4 (9.3 - 32.5)		
Coregonus lavaretus				0.2 (0.2 - 0.3)		3.6 (3.3 - 9.5)	3.9 (2.5 - 9.6)	6.4 (4.0 - 12.9)		10.9 (10.8 - 27.7)	16.1 (10.8 - 53.2)
Abramis brama								3.1 (2.0 - 6.1)		5.2 (5.5 - 13.1)	7.7 (5.5 - 25.2)
Rutilus rutilus						2.2 (2.0 - 4.5)	2.3 (1.5 - 4.5)	3.9 (2.4 - 6.1)		6.6 (6.5 - 13.0)	9.8 (6.5 - 25.0)
Gymnocephalus cernua						3.4 (3.3 - 4.2)	3.7 (2.5 - 4.2)	6.1 (4.0 - 5.7)		10.3 (10.7- 12.2)	15.3 (10.8 - 23.5)
Osmerus eperlanus						2.8 (8.3 - 3.9)	2.9 (6.4 - 4.0)	4.8 (10.1 - 5.3)		8.2 (27.3 - 11.4)	12.2 (27.3 - 22.0)
Gasterosteus aculeatus				0.3 (0.7 - 0.7)		6.4 (11.8 -20.7)	6.7 (9.2 - 21.1)				
Zoarces viviparus						5.0 (6.9 - 9.3)	5.3 (5.4 - 9.4)	8.9 (8.4 - 12.6)		15.1 (22.9 - 27.2)	22.4 (22.9 - 52.2)
Gobius niger				0.5 (0.6 - 1.0)		9.4 (9.4 - 30.3)	10 (7.3 - 30.8)	16.6 (11.5 - 41.2)		28.2 (31.0 - 88.6)	
Neogobius melanostomus						19.0 (16.0 - 30.5)	20.1 (12.4 - 31.0)	33.4 (19.5 -41.5)		56.8 (52.9 - 89.3)	
Pomatoschistus minutus				0.9 (0.5 - 2.0)		16.2 (8.5 - 59.5)	17.1 (6.6 - 60.5)				
Myoxocephalus quadricornis										3.0 (3.3 - 2.9)	4.4 (3.3 - 5.6)
Perca fluviatilis						0.9 (1.3 - 1.0)		1.7 (1.6 - 1.3)		2.8 (4.2 - 2.9)	4.2 (4.3 - 5.5)
Esox Lucius											2.5 (2.7 - 4.1)
Somateria mollissima											2.3 (2.0 - 4.6)
Phalacrocorax carbo											1.5 (1.0 - 1.9)

Table S7. Biomagnification factors (BMFs) for pelagic predator-prey pairs using median, minimum, and maximum dry weight Hg concentrations (per Eq. 2).

**Predator** / **Prey**	**zooplankton**	***Clupea harengus***	***Salmo salar***	***Coregonus lavaretus***	***Osmerus eperlanus***	***Perca fluviatilis***	***Ammodytes tobianus***	***Coregonus albula***	***Gasterosteus aculeatus***	***Esox lucius***	***Phalacrocorax carbo***	***Haliaeetus albicilla***
**phytoplankton**	0.12 (0.1 - 0.03)											
**zooplankton**		14.4 (6.0 - 88.2)		12.6 (11.8 - 17.2)	16.7 (4.7 - 41.7)		2.1 (3.4 - 2.0)	13.1 (18.8 - 14.0)	7.2 (3.3 - 7.9)			
Clupea harengus			1.6 (6.1 - 0.2)			3.4 (5.0 - 1.9)				5.6 (7.9 - 2.5)	9.6 (21.3 - 5.4)	14.1 (21.4 - 10.4)
Salmo salar												9 (3.5 - 42.2)
Coregonus lavaretus			1.8 (3.1 - 1.3)			3.9 (2.5 - 9.6)				6.4 (4.0 - 12.9)	10.9 (10.8 - 27.7)	16.1 (10.8 - 53.2)
Osmerus eperlanus			1.3 (7.7 - 0.5)			2.9 (6.4 - 4.0)				4.8 (10.1 - 5.3)	8.2 (27.3 - 11.4)	12.2 (27.3 - 22.0)
Perca fluviatilis										1.7 (1.6 - 1.3)	2.8 (4.2 - 2.9)	4.2 (4.3 - 5.5)
Ammodytes tobianus						23.1 (8.9 - 81.6)						
Coregonus albula			1.7 (1.9 - 1.6)			3.7 (1.6 - 11.8)				6.2 (2.5 - 15.8)	10.5 (6.8 - 34.1)	15.5 (6.8 - 65.5)
Gasterosteus aculeatus			3.1 (11.1 - 2.8)			6.7 (9.2 - 21.1)						
Esox lucius												2.5 (2.7 - 4.1)
Phalacrocorax carbo												1.5 (1.0 - 1.9)
